# Supplementary material for: Splice-Junction-Based Mapping of Alternative Isoforms in the Human Proteome
Source: Cell Rep. Author manuscript; Available in PMC 2020 Jan 15. (PMC6961840; doi:10.1016/j.celrep.2019.11.026)
Supplement: 2 [file NIHMS1546469-supplement-2.zip › DF1/PXD006675/PulmonaryArtery/PulmonaryArtery_11_NEXN_RAEQVNEDEENQDTAK.pdf]

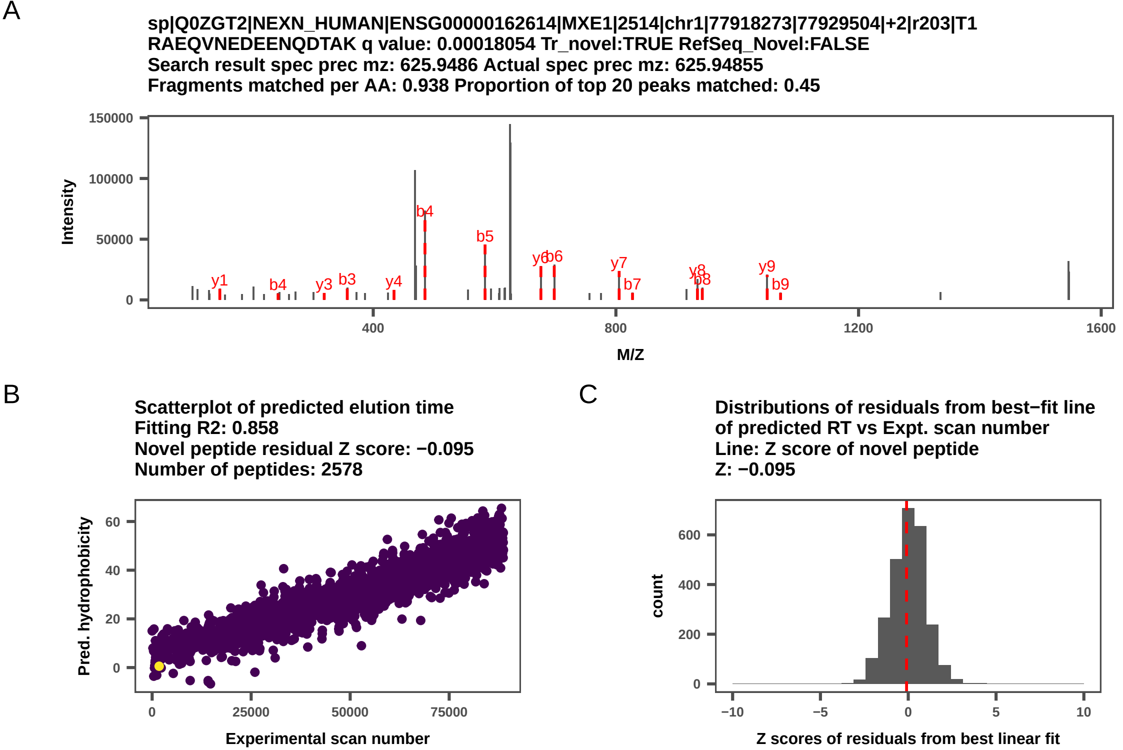
A
sp|Q0ZGT2|NEXN_HUMAN|ENSG00000162614|MXE1|2514|chr1|77918273|77929504|+2|r203|T1
RAEQVNEDEENQDTAK q value: 0.00018054 Tr_novel:TRUE RefSeq_Novel:FALSE
Search result spec prec mz: 625.9486 Actual spec prec mz: 625.94855
Fragments matched per AA: 0.938 Proportion of top 20 peaks matched: 0.45
Intensity
150000
100000
50000
0
y1
b4
y3
b3
y4
b4
b5
y6
b6
y7
b7
y8
b8
y9
b9
400
800
1200
1600
M/Z
B
Scatterplot of predicted elution time
Fitting R2: 0.858
Novel peptide residual Z score: −0.095
Number of peptides: 2578
Pred. hydrophobicity
60
40
20
0
0
25000
50000
75000
Experimental scan number
C
Distributions of residuals from best−fit line
of predicted RT vs Expt. scan number
Line: Z score of novel peptide
Z: −0.095
count
600
400
200
0
−10
−5
0
5
10
Z scores of residuals from best linear fit
